# Moro Orange (*Citrus sinensis* (L.) Osbeck) Extract Mitigates Metabolic Dysregulation, Inflammation, Oxidative Stress, and Adipose Tissue Hyperplasia in Obese Rats

**DOI:** 10.3390/ijms26125727

**Published:** 2025-06-15

**Authors:** Elizandra Gomes Schmitt, Genifer Erminda Schreiner, Laura Smolski dos Santos, Carolina Pereira de Oliveira, Camila Berny Pereira, Silvia Muller de Moura Sarmento, Clovis Klock, Charline Casanova Petry, Elton Luís Gasparotto Denardin, Itamar Luís Gonçalves, Rafael Tamborena Malheiros, Vanusa Manfredini

**Affiliations:** 1 Programa de Pós-graduação em Bioquímica, Universidade Federal do Pampa, BR 472, Km 585, Uruguaiana CEP 97501-970, RS, Brazil; elizandraschmitt.aluno@unipampa.edu.br (E.G.S.); geniferschreiner.aluno@unipampa.edu.br (G.E.S.); laurasantos.aluno@unipampa.edu.br (L.S.d.S.); eltondenardin@unipampa.edu.br (E.L.G.D.); 2 Curso de Enfermagem, Universidade Federal do Pampa, BR 472, Km 585, Uruguaiana CEP 97501-970, RS, Brazil; carolinapdo.aluno@unipampa.edu.br; 3 Curso de Farmácia, Universidade Federal do Pampa, BR 472, Km 585, Uruguaiana CEP 97501-970, RS, Brazil; camilaberny.aluno@unipampa.edu.br; 4 Programa de Pós-Graduação Multicêntrico em Ciências Fisiológicas, Universidade Federal do Pampa, BR 472, Km 585, Uruguaiana CEP 97501-970, RS, Brazil; silviasarmento.aluno@unipampa.edu.br (S.M.d.M.S.); rafaeltmalheiros@gmail.com (R.T.M.); 5 Medicina Diagnóstica, Erechim CEP 99700-000, RS, Brazil; clovisklock@gmail.com (C.K.); casanovacharline@gmail.com (C.C.P.); 6 Departamento de Ciências da Saúde, Universidade Regina Integrada do Alto Uruguai e Missões, Campus de Erechim, Sete de Setembro Avenue, 1621, Erechim CEP 99709-910, RS, Brazil; itamar3141@yahoo.com.br

**Keywords:** *Citrus sinensis*, moro orange, metabolism, interleukins, oxidative stress

## Abstract

This study aimed to evaluate the effects of *C. sinensis* extract, orlistat, and their combination on biochemical, hormonal, inflammatory, and oxidative stress parameters in female rats. The extract was characterized by the presence of citric acid, ferulic acid, and quercetin, along with antioxidant activity. Five experimental groups were established: (1) control; (2) obese; (3) orlistat (1.72 mg/kg); (4) *C. sinensis* extract (7.15 mg/kg); (5) a combination of both treatments. Treatment with the extract, orlistat, or their combination resulted in biochemical parameters (glucose, cholesterol, and triglycerides) that were comparable to the control group and significantly different from the obese group. Notably, only the *C. sinensis* extract alone restored pro-inflammatory cytokine levels (IL-1β, TNF-α, IL-6, IL-8) to values similar to the control. All treatments improved the activity of antioxidant enzymes catalase and glutathione peroxidase, while a significant increase in superoxide dismutase activity was observed only in the extract group. Among the oxidative damage markers, TBARS was the most responsive to treatment, whereas protein carbonylation was less affected. Histological analysis showed that all treatments promoted structural normalization. These results provide a rationale for further pre-clinical and clinical investigations into the use of *C. sinensis* extract as an adjunctive therapy for metabolic disorders.

## 1. Introduction

Obesity has been widely discussed across generations for centuries, both from an esthetic perspective and as a health concern. Numerous studies have explored its causes, effects, and associated factors. Today, obesity is recognized as a major global epidemic and a significant risk factor for various diseases. Estimates indicate that 30% of the world’s population is overweight [1]. In Brazil, data from VIGITEL (Surveillance Survey of Risk and Protection Factors for Chronic Diseases by Telephone Survey) show that in 2019, 55.4% of the population was overweight, and 19.8% was obese. Alarmingly, obesity among young adults (18–24 years) increased by 90%, from 9% in 2022 to 17.1% in 2023 [2].

To study obesity, animal models have been developed to replicate the condition in humans. Diet-induced obesity models are particularly effective in demonstrating the interaction between genetic and environmental factors. Identifying experimental models that closely mimic human obesity and metabolic syndrome is crucial for understanding their pathophysiology and developing more effective treatments [3].

Oxidative stress is a key factor in the progression of various diseases, including diabetes, premature aging, inflammatory disorders, and obesity. As a result, numerous studies have investigated the therapeutic potential of plants and fruits, leveraging traditional knowledge. Regular consumption of fruits and vegetables, rich in antioxidants, has been associated with a reduced incidence of degenerative, neurological, chronic, cardiovascular, and inflammatory diseases [4].

Among fruit-bearing plants, the *Citrus* genus (Rutaceae family) is widely consumed worldwide, with many species studied for their therapeutic properties. These benefits are attributed to bioactive compounds with antioxidant properties, such as phenolic compounds (phenolic acids, coumarins, stilbenes, and flavonoids), as well as vitamins, fiber (pectin), and essential minerals [5].

One promising source of bioactive compounds is the dry extract from Moro orange (*Citrus sinensis* (L.) Osbeck or C. aurantium dulcis) juice. Although research on the biological properties of *C. sinensis* (L.) Osbeck remains limited, there are pre-clinical and clinical studies that suggest its potential effects on obesity and related diseases [6,7], justifying further scientific investigation. Therefore, this study aimed to evaluate the phytochemical profile, in vitro antioxidant activity, biochemical, inflammatory, oxidative stress, and histopathological parameters in obese Wistar rats supplemented with Moro orange extract, assessing its effects on key obesity-related markers.

## 2. Results

### 2.1. Phytochemical Analysis

**Figure 1:**
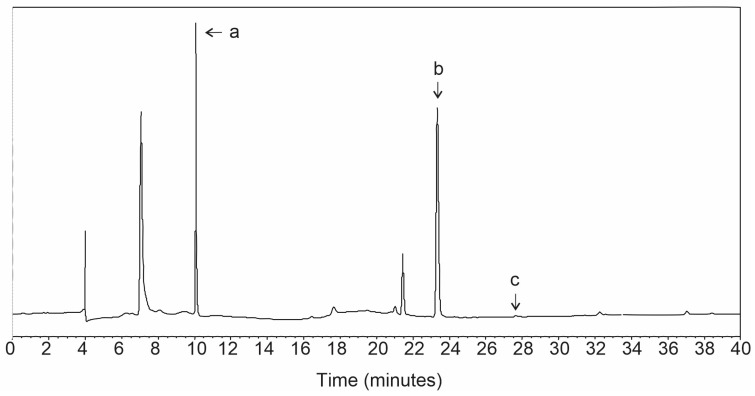
Representative HPLC chromatogram of the *C. sinensis* extract. The mobile phase consisted of water acidified with H_3_PO_4_ to pH 2.9 and acetonitrile, with an injection volume of 20 µL and detection at a wavelength range of 220 nm to 368 nm. Identification of the peaks: a: ferulic acid; b: ascorbic acid; c: quercetin.

Phytochemical analysis revealed the presence of three antioxidant compounds. Ferulic acid (10.64 ± 0.033 mg/L), ascorbic acid (7.48 ± 0.003 mg/L), and quercetin (0.54 ± 0.001 mg/L) were identified. The chromatogram depicting the elution profile of these compounds is presented in Figure 1. The analytical aspects of these quantifications are depicted in Table 1.

### 2.2. In Vitro Antioxidant Activity

The crude extract of *C. sinensis* exhibited a total phenolic content of 5.48 ± 0.14 mg/g. In the ABTS assay, at concentrations of 0.25 mg/mL, it achieved 58.79% inhibition, whereas in the DPPH assay, at concentrations of 0.50 mg/mL, it produced 47.75% inhibition.

### 2.3. In Vivo Effects of Citrus Sinensis on Obese Female Rats

#### 2.3.1. Biochemical Parameters

**Figure 2:**
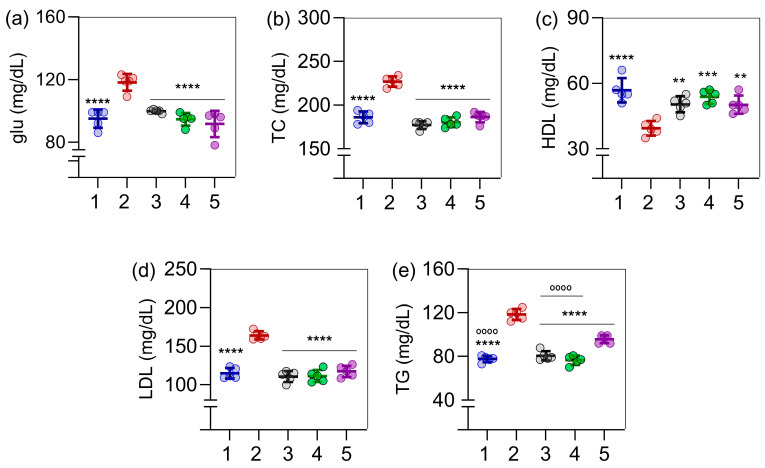
Effects of *C. sinensis* extract and orlistat, administered alone or in combination, on biochemical parameters in obese female rats. In (**a**), the glucose (glu) levels are shown; in (**b**) the total cholesterol (TC) levels; in (**c**), the HDL cholesterol (HDL) levels; in (**d**), the LDL cholesterol (LDL) levels; and in (**e**), the triglyceride levels (TG). The experimental groups were as follows: (1) control; (2) obese (untreated); (3) orlistat; (4) *C. sinensis* extract; (5) orlistat combined with extract. Data are presented as mean ± standard deviation (n = 5). Statistical analysis was performed using one-way ANOVA followed by Tukey’s post hoc test. Symbols indicate statistically significant differences as follows: ** *p* < 0.01, *** *p* < 0.001, **** *p* < 0.0001 (all versus group 2) and °°°° *p* < 0.0001 (all versus group 5).

Glucose (Figure 2a), total cholesterol (Figure 2b), LDL cholesterol (Figure 2d), and triglycerides (Figure 2e) were significantly elevated in obese rats (Group 2) compared to the control group (Group 1), showing the efficacy of the obesity model. In contrast, HDL cholesterol levels were lower in obese rats compared to controls (Figure 2c), with *p* < 0.0001 for all cases. Regarding the biochemical parameters, all were favorably altered in the three experimental treatment groups (groups 3, 4, and 5 were treated with orlistat, *C. sinensis* extract, and their combination, respectively) compared to the obese rats.

#### 2.3.2. Hormonal Parameters

**Figure 3:**
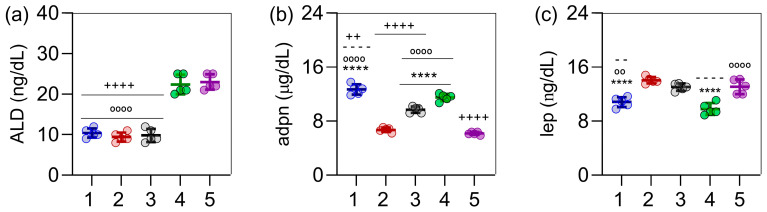
Effect of *C. sinensis* extract and orlistat, alone and in combination, on hormone of obese female rats. In (**a**), aldosterone (ALD) levels are shown; in (**b**), adiponectin (adpn) levels; and in (**c**), leptin (lep) levels. (1) Control; (2) obese rats; (3) orlistat; (4) *C. sinensis* extract; (5) orlistat + extract. (**a**) Aldosterone levels, (**b**) adiponectin levels, and (**c**) leptin levels. Data are expressed as mean ± standard deviation (n = 5). Symbols indicate statistically significant differences as follows: **** *p* < 0.0001 (all versus group 2); − − *p* < 0.01, − − − − *p* < 0.0001 (all versus group 3); ++ *p* < 0.01, ++++ *p* < 0.0001 (all versus group 4); and °° *p* < 0.01, °°°° *p* < 0.0001 (all versus group 5).

Regarding aldosterone levels, groups 4 (extract) and 5 (extract + orlistat) exhibited an increase in aldosterone concentrations compared to groups 1 (control) and 2 (obese rats), as shown in Figure 3a. Adiponectin levels were higher in groups 3 (orlistat) and 4 (extract) compared to the obese rats, while obesity induction led to a decrease in adiponectin levels (Figure 3b). Notably, the group treated with the extract showed the lowest leptin levels, significantly differing from the obese rats (Figure 3c). Furthermore, in terms of adiponectin and leptin, the extract-treated group presented levels comparable to those of the control group.

#### 2.3.3. Inflammatory Parameters

**Figure 4:**
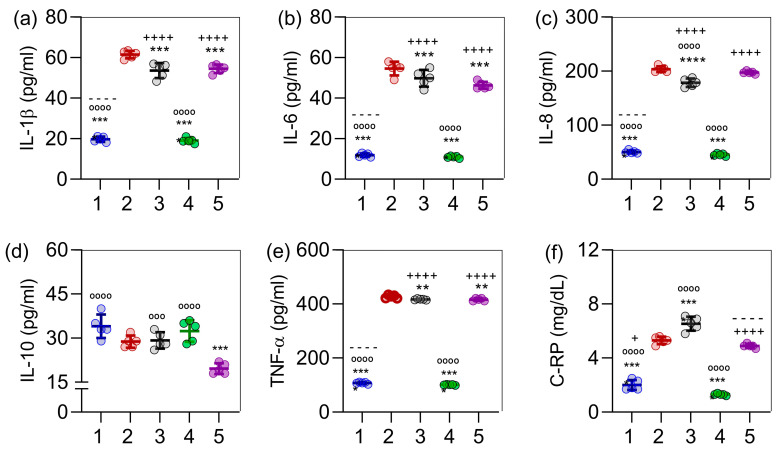
Effect of *C. sinensis* extract and orlistat, alone and in combination, on inflammatory parameters of obese female rats. In (**a**), IL-1β levels are shown; in (**b**), IL-6 levels; in (**c**), IL-8 levels; in (**d**), IL-10 levels; in (**e**), TNF-α levels; and in (**f**), C-reactive protein levels. (1) Control; (2) obese rats; (3) orlistat; (4) *C. sinensis* extract; (5) orlistat + extract. The data are presented as mean ± standard deviation (n = 5). Symbols indicate statistically significant differences as follows: ** *p* < 0.01, *** *p* < 0.001, **** *p* < 0.0001 (all versus group 2); − − − − *p* < 0.0001 (all versus group 3); + *p* < 0.05, ++++ *p* < 0.0001 (all versus group 4); and °°° *p* < 0.001, °°°° *p* < 0.0001 (all versus group 5).

The induction of obesity (group 2) led to a significant increase in all monitored inflammatory interleukins compared to the control group (group 1), as observed for IL-1β (Figure 4a), IL-6 (Figure 4b), IL-8 (Figure 4c), TNF-α (Figure 4e), and C-reactive protein (Figure 4f), with *p* < 0.0001 in all cases. The most pronounced reduction in these inflammatory markers among obese rats was observed in group 4 (treated with *C. sinensis* extract), also with *p* < 0.0001 in all cases. Overall, rats treated with orlistat (group 3) exhibited a less marked decrease in inflammatory cytokines, while the combination of *C. sinensis* extract and orlistat (group 5) resulted in a more moderate reduction. Regarding IL-10 levels (Figure 4d), the lowest value was observed in group 5, which received the combination therapy. Changes in IL-10 were less pronounced; a slight decrease was found in obese rats, while treatments with orlistat and the extract effectively restored IL-10 levels to those of the control group.

#### 2.3.4. Oxidative Stress Parameters

**Figure 5:**
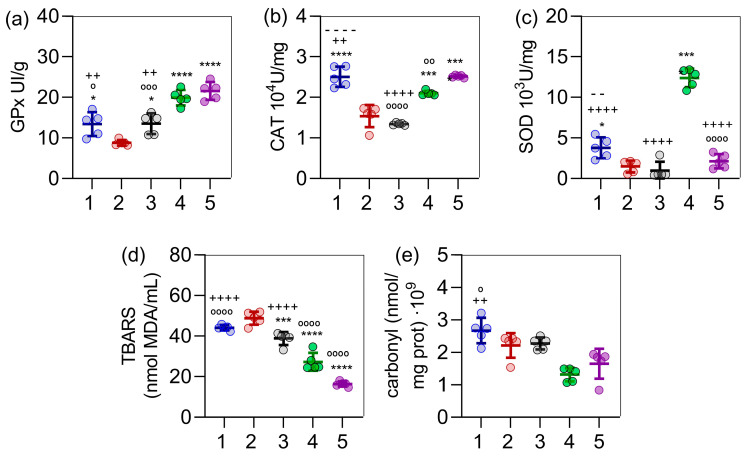
Effect of the experimental protocols—control (group 1), obesity induction (group 2), orlistat treatment (group 3), C. sinensis extract treatment (group 4), and the combination of C. sinensis extract with orlistat (group 5)—on antioxidant enzyme activity and oxidative damage markers in obese female rats. In (**a**), glutathione peroxidase (GPx) levels are shown; in (**b**), catalase (CAT) levels; in (**c**), superoxide dismutase (SOD) levels; in (**d**), thiobarbituric acid reactive substances (TBARS) levels; and in (**e**), protein carbonylation levels. Data are presented as mean ± standard deviation (n = 5). Statistical analysis was performed using one-way ANOVA followed by Tukey’s post hoc test. Symbols indicate statistically significant differences as follows: * *p* < 0.05, *** *p* < 0.001, **** *p* < 0.0001 (all versus group 2); − − *p* < 0.01, − − − − *p* < 0.0001 (all versus group 3); ++ *p* < 0.01, ++++ *p* < 0.0001 (all versus group 4); and ° *p* < 0.05, °° *p* < 0.01, °°° *p* < 0.001, °°°° *p* < 0.0001 (all versus group 5).

Obese rats exhibited significantly lower levels of glutathione peroxidase (GPx, Figure 5a), catalase (CAT, Figure 5b), and superoxide dismutase (SOD, Figure 5c) compared to the control group. Among the treatment groups, group 4 (*C. sinensis* extract) showed the most pronounced difference from group 2 (obese rats) across all three enzymes, with *p* < 0.0001 for GPx and SOD (Figure 5a,c) and *p* < 0.001 for CAT (Figure 5b). Furthermore, for GPx and CAT, treatment with the extract alone or in combination with orlistat (groups 4 and 5) restored these antioxidant markers to levels comparable to or exceeding those of the control group (group 1). Regarding SOD, the extract treatment (group 4) significantly increased its levels beyond those of the control, whereas the combination of extract and orlistat (group 5) resulted in SOD levels that were not significantly different from the control group. Analysis of oxidative damage markers revealed that obesity induction exerted only modest effects, whereas all treatments elicited reductions in both TBARS (Figure 5d) and protein carbonylation (Figure 5e).

#### 2.3.5. Adipose Tissue

**Figure 6:**
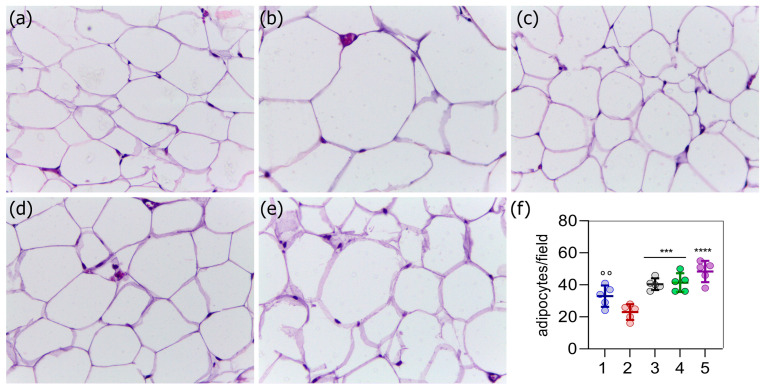
Effect of *C. sinensis* extract on the histology of visceral fat in obese Wistar rats across five experimental groups (**a**–**e**). Control group (**a**), obese group (**b**), group supplemented with orlistat^®^ (**c**), group supplemented with *C. sinensis* extract (**d**), and group supplemented with *C. sinensis* extract + orlistat^®^ (**e**). Optical microscopy performed using a Leica microscope at 40× magnification. Figure (**f**) shows the number of adipocytes per microscope field for each experimental group, where the data are represented as mean ± standard deviation and compared using ANOVA followed by the Tukey test (n = 5). Symbols indicate statistically significant differences as follows: *** *p* < 0.001, **** *p* < 0.0001 (all versus group 2); and °° *p* < 0.01 (all versus group 5).

Adipose tissue samples from the rats were collected and analyzed using optical microscopy to confirm the induction of obesity and to assess the effects of the treatments. Obese rats (Figure 6b) exhibited larger adipocytes compared to those in the control group (Figure 6a). All treatments—including orlistat (Figure 6c), the plant extract (Figure 6d), and their combination (Figure 6e)—resulted in a reduction in adipocyte size, reaching aspects similar to the control group. This effect was further supported by an increased number of adipocytes per microscopic field, as observed under optical microscopy (Figure 6f). Obese rats showed a lower number of adipocytes per field in comparison to the control group (*p* = 0.0742), and significantly lower counts compared to all treated groups: orlistat (*p* = 0.0008), extract (*p* = 0.0004), and the combination treatment (*p* < 0.0001).

### 2.4. Overview of Biochemical, Hormonal, Inflammatory, and Oxidative Stress Parameters

**Figure 7:**
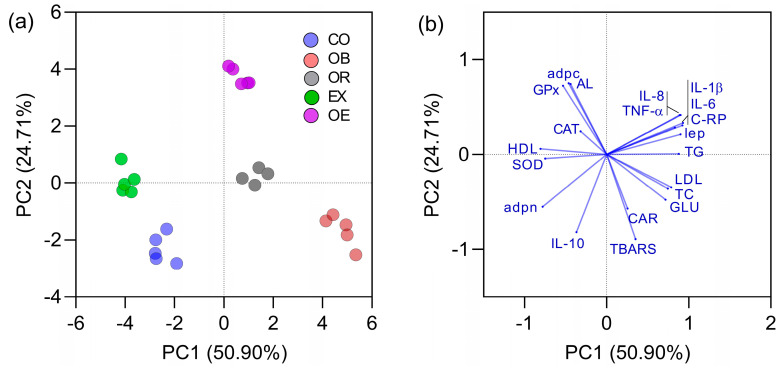
Principal component analysis (PCA) of the 20 variables monitored across experimental groups: (**a**) distribution of individual animals along the first two principal components (PC1 and PC2); (**b**) vector plot illustrating each variable’s contribution to group clustering and the interrelationships among all variables. Abbreviations: adpc, adipocytes per field; Adpn, adiponectin; Lep, leptin; AL, aldosterone; SOD, superoxide dismutase; GPx, glutathione peroxidase; CAT, catalase; TBARS, thiobarbituric acid-reactive substances; CAR, protein carbonylation; IL, interleukins; TNF-α, tumor necrosis factor-alpha; GLU, glucose; TC, total cholesterol; LDL, low-density lipoprotein cholesterol; HDL, high-density lipoprotein cholesterol; TG, triglycerides. CO: control, group 1; OB: obese, group 2; OR: orlistat, group 3; EX: extract, group 4; OE: orlistat + extract, group 5.

Aiming to reduce data dimensionality, a principal component analysis (PCA) was performed: the first two components (PC1 and PC2) together accounted for over 60% of the total variance. The projection of individual animals onto the PC1–PC2 plane (Figure 7a) yielded five well-defined clusters corresponding to the experimental groups. As expected, obese rats (group 2) were localized in the right-hand quadrant and characterized by elevated glucose, total cholesterol, LDL cholesterol, and triglyceride levels. They also displayed a pronounced pro-inflammatory profile—markedly increased IL-1β, IL-6, IL-8 and TNF-α—and lay in the direction of the vectors for those cytokines. Notably, the control group and the extract-treated group clustered closely together, exhibiting nearly identical PC1 and PC2 scores; this mirrors our earlier findings that only the C. sinensis extract restored inflammatory markers to control-like levels, whereas obese animals, with or without orlistat, retained high interleukin concentrations.

The vector plot (Figure 7b) further validates these relationships: TBARS and protein carbonylation vectors form an acute angle (indicating a strong positive correlation), whereas the anti-inflammatory cytokine IL-10 vector lies nearly opposite of the pro-inflammatory interleukin vectors (angle ≈ 180°), signifying an inverse correlation. An additional inverse relationship is evident between HDL cholesterol and the cluster of total cholesterol, LDL cholesterol, and triglycerides.

In addition, no marked adverse effects were observed in any of the five experimental groups, reflecting the safety profile of the extract alone and in combination with orlistat.

## 3. Discussion

Blood oranges of the Moro variety (*Citrus sinensis* L. Osbeck) have been extensively studied for their high levels of anthocyanins, ascorbic acid, and hydroxycinnamic acids [8,9,10]. Our phytochemical analysis corroborates these findings, revealing prominent peaks corresponding to ferulic acid, ascorbic acid, and quercetin. Another study identified ferulic acid as the predominant hydroxycinnamic compound in several orange cultivars [11], a result echoed by Ye et al. (2023), who demonstrated its robust antioxidant activity across multiple assays [12]. In obesity research, ferulic acid has been shown to modulate various physiological parameters, including body mass regulation [8]. Oryzanol and ferulic acid exerted hypolipidemic and antioxidant effects, suggesting a potential role for these phenolics in obesity management [13]. Salazar-Lopez and collaborators (2017) demonstrated that 2 g/kg of ferulic acid in Wistar rats on a diet rich in lard for 8 weeks resulted in a glycemic and homeostatic model assessment index of insulin resistance (HOMA-IR) reduction and presented antiadipogenic properties [14].

Biochemical evaluation confirmed the efficacy of obesity induction via a cafeteria-style diet: group 2 (obese controls) exhibited significantly altered metabolic markers compared to group 1 (lean controls). Both Orlistat^®^ supplementation and treatment with the Moro orange extract effectively ameliorated obesity-related dysregulation in Wistar rats. Specifically, treated animals maintained blood glucose levels comparable to lean controls. Furthermore, groups 3 (Orlistat^®^), 4 (extract), and 5 (combination) all showed significant improvements in total cholesterol, LDL, HDL, and triglyceride levels relative to the obese control group, highlighting the promise of alternative dietary interventions for mitigating obesity’s deleterious effects.

An investigation documented that Moro orange consumption enhances insulin sensitivity and lowers serum triglycerides and cholesterol, findings that align with our observations of the *Citrus sinensis* extract’s beneficial lipid-modulating properties [9]. Furthermore, Moro orange juice intake regulated metabolic and biochemical parameters in obese rats, reinforcing the extract’s therapeutic potential [8]. The dietary supplementation with 5% *Citrus japonica* (kinkan) improved lipid profiles and attenuated oxidative damage in hypercholesterolemic rats [15], while *Citrus aurantium* supplementation reduced serum triglyceride and LDL cholesterol levels [16]. Regarding orlistat, in a randomized clinical trial conducted with overweight and obese men, administration of orlistat at a dose of 120 mg three times per week did not result in significant changes in serum glucose levels or lipid profile when compared to the control group [17].

Hormonal analyses revealed an increase in aldosterone in extract-supplemented groups, warranting further investigation. Notably, both adiponectin and leptin levels improved most markedly in group 4, indicating that the C. sinensis extract suppressed leptin secretion in obese rats. This is consistent with Wu et al. (2018), who observed that anthocyanidins from blackberry and blueberry juices inhibited leptin expression in obese rodents [18]. Another investigation demonstrated that ferulic acid, in addition to modulating glucose metabolism, significantly reduced serum leptin levels and increased adiponectin concentrations [19].

Regarding inflammatory cytokines, group 4 exhibited a reduction in pro-inflammatory interleukins (IL-1β, IL-6, IL-8, and TNF-α) and an elevation in the anti-inflammatory interleukin IL-10, relative to the obese control group. These data demonstrate the extract’s capacity to restore cytokine balance and attenuate inflammation. Oliveira et al. (2020) reported elevated pro-inflammatory adipocytokines—such as angiotensinogen, TNF-α, IL-6, leptin, MCP-1, and resistin—in obese individuals; in our study, extract-treated rats approached control levels for these markers [20]. Others investigations similarly found that Moro orange juice intake improved inflammatory and biochemical parameters in human subjects [21]. Another study demonstrated that the Moro orange phytocomplex, particularly its anthocyanin content, has the potential to reduce inflammation, oxidative stress, and lipid accumulation in adipocytes [22].

Enzymatic antioxidants—catalase (CAT), superoxide dismutase (SOD), and glutathione peroxidase (GPx)—showed enhanced activity in the extract-treated animals versus obese controls. Nunes (2023) reported analogous increases in SOD, CAT, and GPx activities following *Citrus maxima* (pummelo) extract supplementation [7], and Wu et al. (2018) observed similar antioxidant enzyme upregulation with berry anthocyanidins [18]. Moro orange exhibits potent antioxidant and cytoprotective effects under various dietary conditions, consistent with our findings of decreased protein carbonylation and lipid peroxidation in blood and liver tissues of extract-treated groups [9]. In recent years, oxidative stress has been recognized as a key contributor to the progression of various pathologies, including cancer. Reactive oxygen species play a damaging role in cells; however, antioxidant compounds and enzymes act as protective agents against oxidative damage [23].

Over the past decade, our understanding of oxidative stress has expanded significantly. It is now frequently viewed as an imbalance rooted not only in environmental factors but also in genetic mechanisms and the regulation of gene expression. Central to this emerging perspective is the transcription factor known as the nuclear factor (erythroid-derived 2)-like 2 (Nrf2). Often referred to as the master regulator of the antioxidant response, Nrf2 modulates the expression of hundreds of genes—not only those involved in classic antioxidant defense but also genes regulating immune and inflammatory responses, tissue remodeling and fibrosis, carcinogenesis and metastasis, and even cognitive function [24].

In the adipose tissue, obese controls exhibited significant adipocyte hyperplasia, whereas groups 3, 4, and 5 showed normalization toward the lean phenotype. Nunes et al. (2023) similarly demonstrated that *Citrus maxima* extract prevents hepatic fat accumulation in a nonalcoholic fatty liver disease model [7]. Magalhães et al. (2020) reported that Moro orange juice reduced adipocyte size and body mass after several weeks of treatment [8], while Wu et al. (2018) linked reduced adiposity and leptin production to berry anthocyanin supplementation [18].

Future studies should extend treatment duration to assess the long-term histological impact on liver and adipose tissues. Interestingly, group 4 consistently outperformed both Orlistat^®^ and combination treatments across multiple parameters, approximating lean control values in several analyses. Al-Kuraishy and Al-Garreb (2016) observed a similarly favorable cardiometabolic profile when combining Garcinia cambogia with Orlistat^®^ versus medication alone, underscoring the potential synergy between phytochemicals and conventional therapies [25].

## 4. Materials and Methods

### 4.1. Chemicals

All chemicals used were of analytical grade. Analytical standards were purchased from Sigma-Aldrich Chemical Corporation (St. Louis, MO, USA) and Merck (Darmstadt, Germany). Orlistat^®^ (Roche, São Paulo, Brazil) and *Citrus sinensis* (L.) Osbeck extract (Galena, São Paulo, Brazil) were obtained from a local compounding pharmacy. According to the manufacturer, the extract contains 4.5% (*w*/*w*) total anthocyanins.

### 4.2. Extract Analysis

#### 4.2.1. Extraction

The vegetal extract was dissolved in 10 mL of methanol: water 1:1 and submitted to an ultrasound bath for 30 s. Afterwards, the solution was filtered in a PTFE filter (0.22 µm × 13 mm) and transferred to a HPLC vial.

#### 4.2.2. Chromatographic Analysis

The major compounds present in the extract were quantified using high-performance liquid chromatography with photodiode array detection (HPLC-PDA), with ultraviolet detection ranging from 200 to 400 nm. Separation was performed on a C18 column (Phenomenex (Torrance, CA, USA) KJ0-42B2, 5 µm; 4.6 × 250 mm) as the stationary phase. The mobile phase consisted of a mixture of phosphoric acid (H_3_PO_4_) adjusted to pH 2.9 and acetonitrile in a gradient starting with acetonitrile 5% and reaching 95% at t = 35 min at a rate of 0.33 mL/min.

Calibration curves were prepared using chlorogenic acid, caffeic acid, p-coumaric acid, ferulic acid, rutin, and quercetin at concentrations of 10.0, 5.0, 2.0, 1.0, 0.1, and 0.05 mg/L. Detection wavelengths were set at 220 nm for rutin, 320 nm for chlorogenic acid, caffeic acid, p-coumaric acid, and ferulic acid, and 368 nm for quercetin.

#### 4.2.3. Phenolic Determination

The total phenolic content was determined using the Folin–Ciocalteu method [26]. A 500 mg sample of *C. sinensis* extract was diluted in 1 mL of deionized water. From this solution, 125 μL was mixed with 500 μL of deionized water and 125 μL of Folin–Ciocalteu reagent (1 M). The mixture was left to rest for 6 min before adding 1.25 mL of sodium carbonate (Na_2_CO_3_) solution at 7% and 2 mL of deionized water. The reaction mixture was allowed to stand for an additional 90 min, after which the absorbance was measured at 760 nm using a UV-1800 spectrophotometer (Shimadzu, Kyoto, Japan). A calibration curve was constructed using gallic acid in concentrations ranging from 0.025 to 0.5 mg/mL. The total polyphenol content was expressed as mg of gallic acid equivalents (GAEs) per gram of extract. The regression equation obtained was y = 64.473x − 0.0642, with a high coefficient of determination (r^2^ = 0.9912), indicating a good fit.

#### 4.2.4. In Vitro Antioxidant Determination

The ABTS assay was conducted following previously established protocols [27]. Six concentrations of *C. sinensis* extract (ranging from 0.25 to 5 mg/mL) were prepared, with ascorbic acid used as the positive control. A volume of 1000 μL of ABTS solution (42.77 μM) was added to each assay tube. The samples were allowed to stand for 6 min before measuring absorbance at 734 nm using a UV-1800 spectrophotometer (Shimadzu, Kyoto, Japan). The antioxidant activity was expressed as the concentration required to reduce 50% of the ABTS radical (IC_50_).

The DPPH radical scavenging activity was assessed following the protocol previously reported [28]. For the blank control, 10 μL of Milli-Q water was pipetted in duplicate into test tubes. For the sample tubes, 10 μL of each of the six concentrations of the *C. sinensis* extract was pipetted in duplicate. Similarly, for the positive control, 10 μL of each of the six concentrations of ascorbic acid was added in duplicate. All tubes were protected from light, and 1000 μL of a 50.5 μM diluted DPPH solution was added to each tube, with 30 s intervals between additions (processing a maximum of 12 samples at a time). The tubes were then incubated at 30 °C in the dark for 30 min. Following incubation, absorbance readings were taken at 517 nm using a spectrophotometer, with 30 s intervals between samples. The spectrophotometer was calibrated with analytical-grade methanol using a plastic cuvette. The DPPH radical scavenging capacity of the compounds was calculated based on the IC_50_ value.

### 4.3. Animals

A total of 25 female Wistar rats, aged 45 days, were used in this study. The animals were obtained from the vivarium of the Federal University of Pampa (UNIPAMPA). They were acclimated for 15 days before the start of the experimental protocols and maintained under controlled conditions, including a 12 h light–dark cycle, temperature, and humidity control. All animals had free access to food and water (ad libitum). The experiments were conducted in accordance with the ethical guidelines for biomedical research established by the Brazilian Societies of Experimental Biology and were approved by the Ethics Committee for Animal Use of UNIPAMPA, under protocol number 07/2022.

### 4.4. Obesity Induction

After the acclimatization period, the animals were weighed, and the experimental protocols were initiated when they reached a body weight between 180 and 200 g at 63 days of age. The animals were fed a crushed commercial formulation supplemented with pâté, French fries, bacon, salted biscuits, and chocolate in a ratio of 2:1:1:1:1:1, with water provided ad libitum. The 45 g diet sample provides 183.7 kcal, and is composed of 5.39 g of protein, 18.60 g of carbohydrates, 10.76 g of total fat (including 4.07 g of saturated fat and 0.017 g of trans fat), 0.56 g of dietary fiber, and 246.73 mg of sodium [29]. The rats in the control group received a standard diet provided by Presence Labina, suitable for rats, mice, and other rodents. Throughout the entire protocol, the diets remained unchanged. The diet was administered once daily, always at the same time, for a period of nine consecutive weeks. The animals were weighed once a week, and both abdominal circumference and naso-anal length were measured.

### 4.5. Experimental Layout

After obesity induction, the 25 female rats were divided into experimental groups. The administration of the extract or orlistat was performed once daily at the same time by gavage. The experimental groups were as follows: (1) control group, which received a 0.9% saline solution; (2) obese group, which also received a 0.9% saline solution; (3) obese rats treated with orlistat at a dose of 1.72 mg/kg, equivalent to the human dose of 120 mg; (4) obese rats treated with *C. sinensis* extract at a dose of 7.15 mg/kg, corresponding to the human dose of 500 mg; (5) obese rats treated with both orlistat and *C. sinensis* extract at the same doses used in groups (3) and (4).

At the end of the experiment, the rats were anesthetized with ketamine (80 mg/kg) and xylazine (10 mg/kg) administered via intraperitoneal injection. Total blood was collected through cardiac puncture and subsequently fractionated into tubes containing EDTA or a gel separator to obtain whole blood, plasma, and serum through centrifugation. Adipose and hepatic tissues were also collected for analysis.

### 4.6. Analytical Determinations

The glycemic and lipid profiles (total cholesterol, LDL, HDL, and triglycerides) were determined using serum samples and commercial kits with the Labtest (Minas Gerais, Lagoa Santa, Brazil) and Chem Well T equipment (Labtest, Minas Gerais, Brazil). For the analysis of inflammatory markers (IL-1β, IL-6, IL-8, IL-10, TNF-α, and C-RP) and hormonal parameters (leptin, adiponectin, and aldosterone), 500 µL of serum stored at −80 °C was used. These analyses were performed by the ELISA method using with kits from Thermo Fisher Scientific (São Paulo, Brazil).

In addition, the antioxidant enzymes catalase (CAT), glutathione peroxidase (GPx), and superoxide dismutase (SOD) were quantified. CAT activity was determined using a previously published protocol [20], while SOD and GPx activities were measured using commercial kits from RANSOD (RANDOX, São Paulo, Brazil) and RANSEL (RANDOX, São Paulo, Brazil), respectively.

To evaluate oxidative damage to biomolecules, thiobarbituric acid-reactive substances (TBARS) were quantified in plasma samples according to the method described by Ohkawa (1979) [30]. Protein carbonylation was also measured in plasma samples following the method developed by Levine et al. (1990) [31].

### 4.7. Histological Analysis

Adipose tissue samples were stored in 1% formalin until processing, which included fixation, paraffin embedding, and sectioning with a microtome. The slides were stained following the Hematoxylin and Eosin (H&E) protocol [32]. Images were captured using a Leica DM50 microscope (Wetzlar, Germany) equipped with a 10-megapixel digital camera at 40× magnification. Adipocyte counts per field were also performed at the same magnification.

### 4.8. Data Analysis

All the parameters were expressed as mean ± standard deviation and the normality of the data was investigated by the Shapiro–Wilk test. Afterwards, the mean of the parameters was compared among the five experimental groups using variance analysis followed by the Tukey test. Principal component analysis (PCA) was conducted to provide an integrated overview of the treatment effects across all investigated parameters. All the *p* values lower than 0.05 were considered as significative. The analysis was performed using the GraphPad Prism 9.2.

## 5. Conclusions

The *C. sinensis* extract significantly improved the biochemical profile of obese rats, modulating glucose, lipid parameters (total cholesterol, HDL, LDL, and triglycerides), and oxidative stress markers. Notably, only the extract was capable of fully restoring pro-inflammatory cytokine levels (IL-1β, TNF-α, IL-6, and IL-8) to values similar to those observed in healthy controls, suggesting a unique immunomodulatory role not achieved by orlistat or the combined therapy. Furthermore, the extract enhanced the activity of key antioxidant enzymes (SOD, CAT, and GPx) and more effectively mitigated oxidative damage to proteins and lipids than orlistat. Although all treatments normalized adipocyte morphology, the broader spectrum of biochemical and inflammatory improvements observed with *C. sinensis* underscores its multifaceted therapeutic potential.

These findings position *C. sinensis* extract as a promising candidate for the development of integrative anti-obesity interventions, particularly in strategies targeting both metabolic dysregulation and chronic inflammation. Future studies, including clinical trials, are warranted to validate these effects and elucidate the underlying molecular mechanisms.

## Figures and Tables

**Table 1:** Analytical parameters for analyte quantification.

Analyte	Equation	R^2^	Detection Limit	Quantification Limit
Ascorbic acid	y = 164,172x + 62,032	0.9872	0.006611	0.041779
Ferrulic acid	y = 336,955x − 97,822	0.9933	0.015962	0.239042
Quercetin	y = 240,945x − 63,721	0.9954	0.003653	0.10433

## Data Availability

The data that support the results of this study are available from the corresponding author request.
